# Multi-Organ Expression Profiling Uncovers a Gene Module in Coronary Artery Disease Involving Transendothelial Migration of Leukocytes and LIM Domain Binding 2: The Stockholm Atherosclerosis Gene Expression (STAGE) Study

**DOI:** 10.1371/journal.pgen.1000754

**Published:** 2009-12-04

**Authors:** Sara Hägg, Josefin Skogsberg, Jesper Lundström, Peri Noori, Roland Nilsson, Hua Zhong, Shohreh Maleki, Ming-Mei Shang, Björn Brinne, Maria Bradshaw, Vladimir B. Bajic, Ann Samnegård, Angela Silveira, Lee M. Kaplan, Bruna Gigante, Karin Leander, Ulf de Faire, Stefan Rosfors, Ulf Lockowandt, Jan Liska, Peter Konrad, Rabbe Takolander, Anders Franco-Cereceda, Eric E. Schadt, Torbjörn Ivert, Anders Hamsten, Jesper Tegnér, Johan Björkegren

**Affiliations:** 1 The Computational Medicine Group, Atherosclerosis Research Unit, Department of Medicine, Karolinska Institutet, Stockholm, Sweden; 2 Department of Computational Biology, Linköping Institute of Technology, Linköping University, Linköping, Sweden; 3 Clinical Gene Networks AB, Karolinska Science Park, Stockholm, Sweden; 4 Rosetta Inpharmatics, Merck, Seattle, Washington, United States of America; 5 South African National Bioinformatics Institute (SANBI), University of the Western Cape, Cape Town, South Africa; 6 Computational Bioscience Research Center (CBRC), King Abdullah University of Science and Technology (KAUST), Thuwal, Kingdom of Saudi Arabia; 7 Department of Clinical Sciences, Danderyd Hospital, Karolinska Institutet, Stockholm, Sweden; 8 Cardiovascular Genetics Group, Atherosclerosis Research Unit, Department of Medicine, Karolinska Institutet, Stockholm, Sweden; 9 Massachusetts General Hospital (MGH) Weight Center and Department of Medicine, Harvard Medical School, Boston, Massachusetts, United States of America; 10 Department of Environmental Medicine, Karolinska Institutet, Stockholm, Sweden; 11 Department of Clinical Physiology, Stockholm Söder Hospital, Karolinska Institutet, Stockholm, Sweden; 12 Department of Thoracic Surgery and Anesthesiology, Karolinska University Hospital, Stockholm, Sweden; 13 Department of Molecular Medicine and Surgery, Karolinska Institutet, Stockholm, Sweden; 14 Department of Surgery, Stockholm Söder Hospital, Karolinska Institutet, Stockholm, Sweden; University of Washington, United States of America

## Abstract

Environmental exposures filtered through the genetic make-up of each individual alter the transcriptional repertoire in organs central to metabolic homeostasis, thereby affecting arterial lipid accumulation, inflammation, and the development of coronary artery disease (CAD). The primary aim of the Stockholm Atherosclerosis Gene Expression (STAGE) study was to determine whether there are functionally associated genes (rather than individual genes) important for CAD development. To this end, two-way clustering was used on 278 transcriptional profiles of liver, skeletal muscle, and visceral fat (n = 66/tissue) and atherosclerotic and unaffected arterial wall (n = 40/tissue) isolated from CAD patients during coronary artery bypass surgery. The first step, across all mRNA signals (n = 15,042/12,621 RefSeqs/genes) in each tissue, resulted in a total of 60 tissue clusters (n = 3958 genes). In the second step (performed within tissue clusters), one atherosclerotic lesion (n = 49/48) and one visceral fat (n = 59) cluster segregated the patients into two groups that differed in the extent of coronary stenosis (*P* = 0.008 and *P* = 0.00015). The associations of these clusters with coronary atherosclerosis were validated by analyzing carotid atherosclerosis expression profiles. Remarkably, in one cluster (n = 55/54) relating to carotid stenosis (*P* = 0.04), 27 genes in the two clusters relating to coronary stenosis were confirmed (n = 16/17, *P*<10^−27and−30^). Genes in the transendothelial migration of leukocytes (TEML) pathway were overrepresented in all three clusters, referred to as the atherosclerosis module (A-module). In a second validation step, using three independent cohorts, the A-module was found to be genetically enriched with CAD risk by 1.8-fold (*P*<0.004). The transcription co-factor LIM domain binding 2 (LDB2) was identified as a potential high-hierarchy regulator of the A-module, a notion supported by subnetwork analysis, by cellular and lesion expression of *LDB2*, and by the expression of 13 TEML genes in *Ldb2*–deficient arterial wall. Thus, the A-module appears to be important for atherosclerosis development and, together with LDB2, merits further attention in CAD research.

## Author Summary

The WHO predicts that coronary artery disease (CAD) will become the leading cause of death worldwide in 2010. Currently, major research efforts are focused on understanding the genetics of CAD through multi-center, genome-wide association studies of tens of thousands of patients and controls. Such studies can identify common variants of general importance throughout the entire population, which are likely relatively few. The number of rare genetic variants and variants that act in the context of environmental risk factors for CAD is probably much higher. We performed whole-genome expression analyses in several organs to identify functionally associated genes important for CAD development. We found an atherosclerosis module (A-module) consisting of 128 genes, enriched with genetic risk for CAD, involving transendothelial migration of leukocytes (TEML) and LIM domain binding 2 (LDB2) as its high-hierarchy regulator. Our study design represents a novel way of understanding the molecular underpinnings of CAD, focusing on genome-wide expression sensing both environmental and genetic influences. Investigating the relative enrichment of genetic CAD risk in functional groups (modules and networks) is an alternative approach to extract additional relevant information from genome-wide association studies. The A-module and LDB2 are attractive targets for treatments to modulate TEML and atherosclerosis development.

## Introduction

The mapping of the human genome resulted in new technologies for studying complex diseases such as coronary artery disease (CAD) from a functional genomic perspective. By revealing comprehensive repertoires of molecular activities, these technologies combined with systems biology analyses will pave the way for a more detailed understanding of the complexity underlying common disorders—a prerequisite to advance molecular diagnostics for early identification of disease and to identify central disease pathways for therapies tailored to specific disease mechanisms [1]–[3].

The aim of the Stockholm Atherosclerosis Gene Expression (STAGE) study was to identify functionally associated genes important for CAD using whole-genome expression profiles from multiple organs. To this end, we used a modified version of a two-way clustering approach [4]–[6]. In the first step, the algorithm processed all mRNA signals within one organ to define a number of tissue clusters. The individual genes of the tissue clusters are defined by the level of associations between mRNA signals across all patients. In the second step, the patients are clustered according to the mRNA signals within each tissue cluster to identify signals related to clinical phenotypes. In this study, the clinical endpoint was the extent of coronary atherosclerotic lesions as judged from the degree of coronary stenosis, measured by quantitative coronary angiography (QCA). A secondary hypothesis was to reveal the extent to which any tissue cluster related to coronary stenosis acts in isolation in one organ or across several organs.

A multi-organ biopsy approach is primarily motivated by the nature of CAD development: atherosclerotic diseases are believed to start in adolescence and develop throughout life [7]. The pace of development depends on genetic and environmental risk factors. Of particular importance are metabolic disturbances (e.g. overweight, diabetes and dyslipidemias) that originate in organs central to energy metabolism, including liver, skeletal muscle, and fat deposits. Thus, molecular activities (mirrored by mRNA levels) distant from the actual site of CAD are likely to influence the progression and extent of coronary atherosclerosis.

The STAGE study comprises 114 carefully characterized patients, including a compendium of 278 global gene-expression profiles from five CAD-relevant tissues isolated during coronary artery bypass grafting (CABG). Using a two-way clustering approach, we analyzed this compendium to test our main hypothesis that there are groups of functionally associated genes (rather than individual genes) of importance for CAD and to determine whether those groups of genes act in isolation in each tissue or across several tissues.

## Results

### Exploratory Clustering of Gene-Expression Profiles in the STAGE Cohort

To test the main hypothesis of the study we explored the gene expression profiles of the STAGE cohort. Gene expression profiles could not be obtained from all tissues in all patients of the STAGE cohort (n = 114). Therefore, it was important to examine whether the two subgroups of patients in which gene expression profiles were obtained—66 patients with gene expression profile from visceral fat, liver, and skeletal muscle and 40 in whom expression profiles were also obtained from atherosclerotic and unaffected arterial wall—had similar clinical phenotypes. Indeed, this appeared to be the case (Table 1).

**Table 1:** Basic characteristics of the STAGE cohort.

Characteristics	STAGE					Carotid patients
	Entire cohort	Metabolic expression profiles	*p*-Value	Complete expression profiles	*p*-Value	expression profiles
***n*** ** (% of total)**	114 (100)	66 (58)		40 (35)		25 (100)
**Age, y (mean±SD)**	66±8	66±8		66±8		69±11
**Male, ** ***n*** ** (%)**	102 (89)	59 (89)		37 (93)		15 (60)
**Body-mass index, kg/m^2^ (mean±SD)**	26.6±3.7	26.4±3.9		26.3±3.9		25.3±3.2
**Waist-to-hip ratio (mean±SD)**	0.94±0.06	0.93±0.06		0.93±0.06		0.91±0.07
**Blood pressure, mm Hg (mean±SD)**						
Systolic	141±19	140±19		135±18		150±19
Diastolic	80±9	80±10		78±8		77±9
**Insulin, pmol/L (mean±SD)**	62±47	59±49		61±53		44±16
**Proinsulin, pmol/L (mean±SD)**	5.6±5.7	5.1±5.7		5.5±6.9		4.6±2.4
**HbA1c, % (mean±SD)**	5.2±1.3	5.0±0.7		5.0±0.6		4.8±0.4
**Cholesterol, mmol/L (mean±SD)**						
Total	4.08±1.01	3.97±1.08		3.83±1.02		4.74±1.21
VLDL	0.32±0.25	0.29±0.25		0.26±0.25		0.22±0.17
LDL	2.09±0.79	2.01±0.84		1.87±0.76		2.60±0.90
HDL	1.49±0.29	1.51±0.33		1.54±0.39		1.74±0.48
**Triglycerides, mmol/L (mean±SD)**						
Total	1.41±0.73	1.36±0.70		1.41±0.76		1.23±0.49
VLDL	1.04±0.67	0.97±0.64		0.98±0.68		0.79±0.42
LDL	0.26±0.09	0.27±0.09		0.28±0.09		0.29±0.09
HDL	0.16±0.05	0.17±0.05		0.19±0.06	<0.01	0.20±0.08
**Current smoker, ** ***n*** ** (%)**	8 (7)	4 (6)		2 (5)		1 (4)
**Former smoker, ** ***n*** ** (%)**	70 (61)	42 (64)		25 (63)		18 (67)
**Alcohol consumption, g/week (mean±SD)**	120±96	117±89		124±82		117±106
**Stenosis score (mean±SD)**	-	5.06±2.41		5.37±2.43		NA
**IMT, mm (mean±SD)**	NA	NA		NA		1.24±0.24
**Diabetes mellitus, ** ***n*** ** (%)**	24 (21)	11 (17)		5 (13)	<0.05	2 (8)
Insulin-requiring	23 (20)	9 (14)		5 (13)		1 (4)
**Hyperlipidemia, ** ***n*** ** (%)**	84 (74)	49 (74)		27 (68)		13 (52)
Statins	101 (89)	61 (92)		37 (93)		15 (60)
**Hypertension, ** ***n*** ** (%)**	72 (63)	43 (65)		25 (63)		16 (64)
Betablocker	103 (90)	62 (94)		38 (95)		11 (44)
ACE inhibitors	42 (37)	25 (38)		15 (38)		5 (20)
Thiazide diuretics	0 (0)	0 (0)		0 (0)		1 (4)
Loop diuretics	26 (23)	13 (20)		10 (25)		3 (12)
Calcium-channel blockers	15 (13)	7 (11)		4 (10)		5 (20)

*p*-Values were calculated using unpaired t-tests comparing subgroups in STAGE with the entire STAGE cohort (n = 114). Subgroups are included in the entire cohort.

NA indicates not available. HbA1c, glycated haemoglobin; VLDL, very low density lipoprotein; LDL, low density lipoprotein; HDL, high density lipoprotein; IMT, intima-media thickness; ACE, angiotensin-converting enzyme.

**Figure 1:**
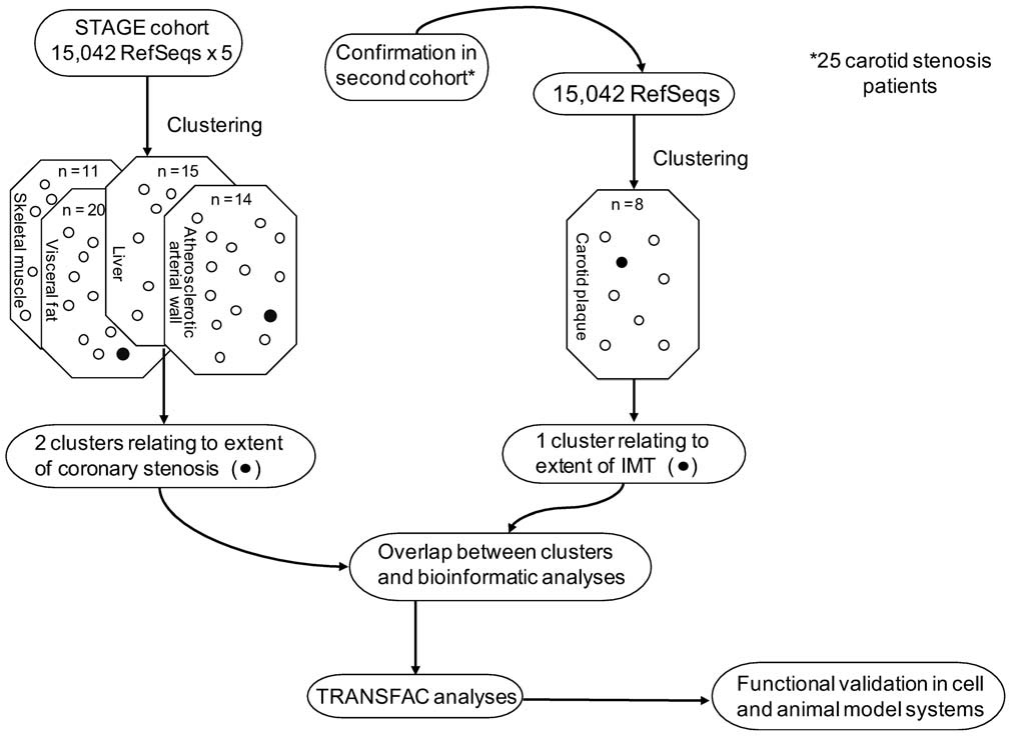
Analytical scheme of multi-organ clustering steps in the STAGE study. Sixty-six gene profiles (15,042 RefSeqs each) from liver, skeletal muscle, and visceral fat and 40 from atherosclerotic aortic wall were clustered by a coupled two-way approach. First, the RefSeqs were clustered according to their average probe signal values on the chip (mRNA level, see figure “clustering”) resulting in 11 skeletal muscle, 20 visceral fat, 15 liver, and 14 atherosclerotic arterial wall clusters together representing 4007 RefSeqs/3958 genes. Second, clustering within each tissue cluster was performed to sort patients by mRNA levels. Clusters that sorted the patients according to extent of coronary stenosis were considered further. To validate these atherosclerosis-related clusters, we performed cluster analysis of 25 gene-expression profiles of carotid atherosclerosis lesions. Of eight clusters representing 903 RefSeqs/894 genes, one segregated patients according to IMT. The extent of overlap between this cluster relating to carotid atherosclerosis and the two clusters relating to coronary atherosclerosis was used as the confirmatory measure. Genetic enrichment and functional gene classifications were then assessed by bioinformatic and TRANSFAC analyses. Animal and cell models were used for functional validation of individual genes.

In the first step of the two-way clustering analysis, mRNA signals of 15,042 Reference Sequence transcripts (RefSeq) were examined in each tissue (Figure 1, Text S1, Figure S1). Importantly, the first step was performed without preconceptions about the extent of coronary atherosclerosis in the CABG patients. Instead, tissue-specific mRNA signals across the patients were analyzed solely to determine whether or not a given RefSeq belonged to a group of functionally associated genes in a tissue cluster. The first clustering step generated 60 tissue clusters representing 4007 RefSeqs/3958 genes (Table S1). Thus, 73% of the RefSeqs or 11,035 RefSeqs (8663 genes) were excluded from further analysis (i.e., the second clustering step). Of these 60 tissue clusters, 15 were identified from the liver gene expression profiles, 11 from skeletal muscle profiles, 20 from visceral fat profiles, and 14 from gene expression profiles of the atherosclerotic arterial wall (Table S1). To assess the repeatability and reliability of these clusters, resampling using Jackknife analysis was performed (Table S1).

**Figure 2:**
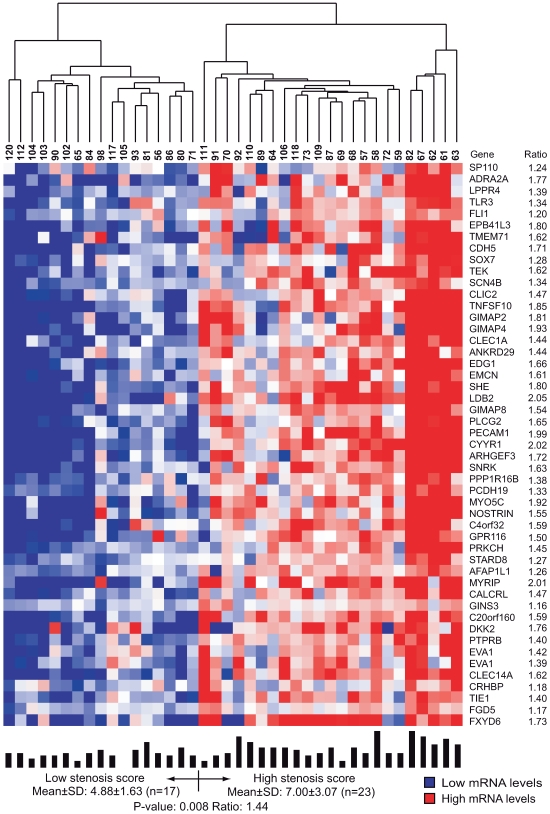
Heat map of an atherosclerotic arterial wall cluster related to coronary stenosis. The cluster was defined by related mRNA levels (indicated by average probe signals on the arrays) and identified as one of fourteen atherosclerotic arterial wall clusters by the second step of coupled two-way clustering of mRNA profiles from STAGE patients (Text S1). Columns represent individual patients, and rows individual RefSeqs with corresponding gene symbols and mRNA ratios of the two patient groups. Above heat map: individual patient numbers, below heat map: bars indicating individual stenosis score together with means ± SD and average ratios in each group and *P*-values for comparing groups. *EVA1* is represented by two RefSeqs.

**Figure 3:**
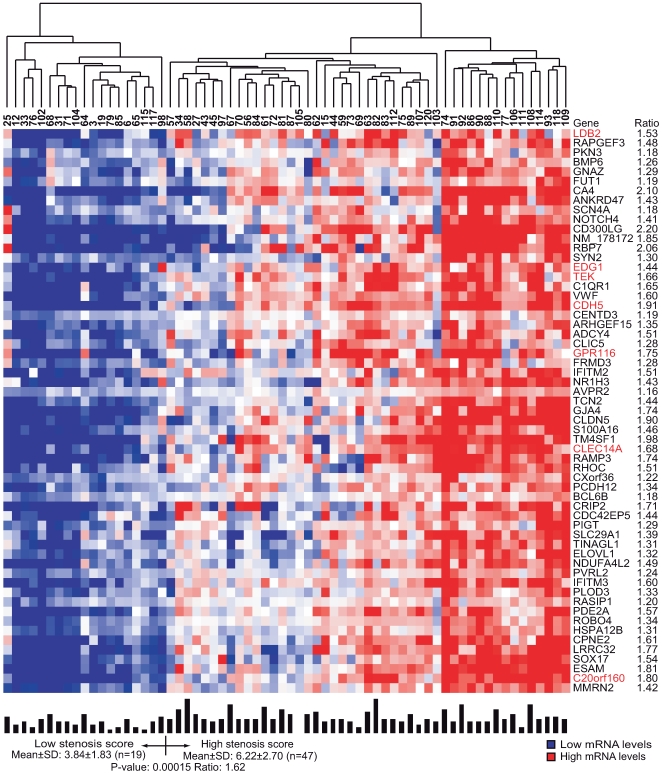
Heat map of a visceral fat cluster related to coronary stenosis. The cluster was defined by related mRNA levels (indicated by average probe signals on the arrays) and identified as one of 20 visceral fat clusters by the second step of coupled two-way clustering of mRNA profiles from STAGE patients (Text S1). Columns represent individual patients, and rows individual RefSeqs with corresponding gene symbols and mRNA ratios of the two patient groups. Above heat map: individual patient numbers, below heat map: bars indicating individual stenosis score together with means ± SD and average ratios in each group and *P*-values for comparing groups. Red highlighting indicates genes also found in the cluster in Figure 2.

In the second step of clustering, the mRNA signals within each of the 60 tissue clusters were used to cluster the patients. The extent of coronary stenosis, determined by QCA, was then compared in the resulting patient groups. Two of the 60 tissue clusters (n = 49 RefSeqs/48 genes, Table S2, (90% CI: 28–49) and n = 59 RefSeqs/genes, Table S3, (90% CI: 38–59), respectively) segregated the patients into groups according to the extent of coronary stenosis: one cluster in atherosclerotic arterial wall and one in visceral fat (*P* = 0.008 (Figure 2) and *P* = 0.00015 (Figure 3), respectively).

To determine whether the identified tissue clusters relating to coronary atherosclerosis are tissue-specific or present in several tissues, we assessed the gene overlap between the atherosclerosis-related clusters in atherosclerotic arterial wall and visceral fat. Seven genes (12%, 14% respectively) were present in both tissue clusters. Although this overlap may appear small, the statistical likelihood of observing an overlap of this size by chance was less than 10^−10^. Thus, this overlap indicates atherosclerosis-related gene activity common to both visceral fat and atherosclerotic arterial wall.

### Confirmatory Clustering of Gene-Expression Profiles of Carotid Lesions

**Figure 4:**
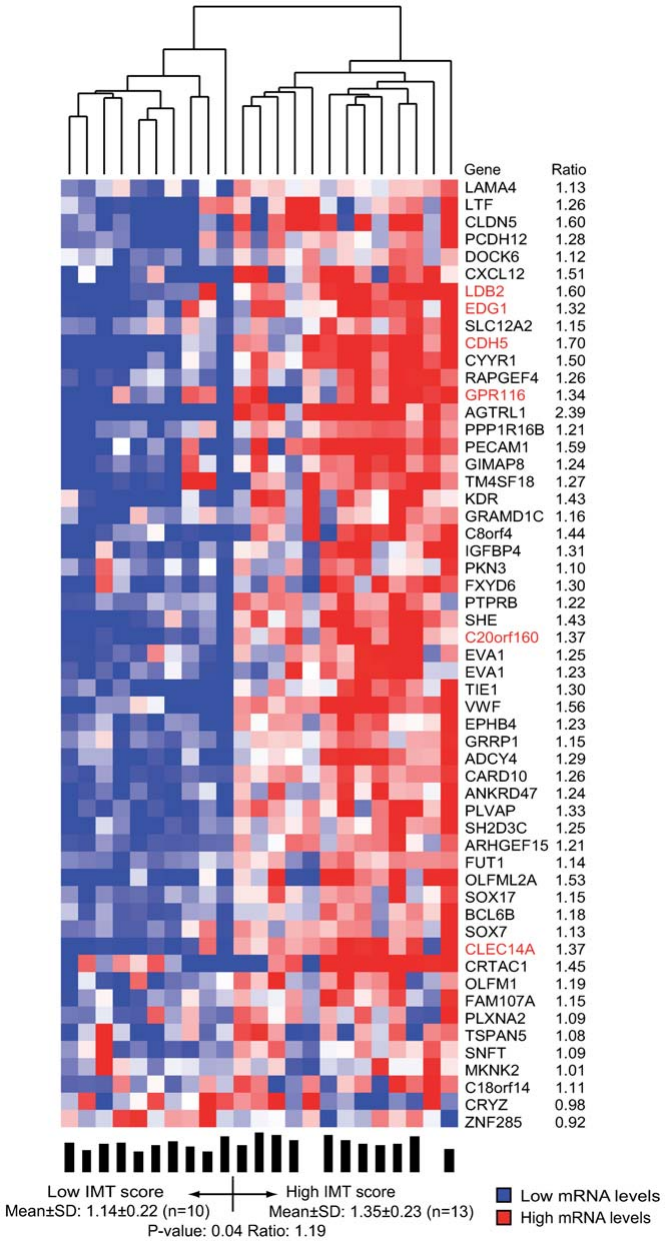
Heat map of a carotid stenosis cluster related to IMT. The cluster was defined by related mRNA levels (indicated by average probe signals on the arrays) and identified as one of eight carotid stenosis clusters by the second step of coupled two-way clustering of mRNA profiles from Carotid Stenosis patients (Text S1). Columns represent individual patients, and rows individual RefSeqs with corresponding gene symbols and mRNA ratios of the two patient groups. Below heat map: bars indicating individual IMT together with means ± SD and average ratios in each group and *P*-values for comparing groups. Red highlighting indicates genes also identified in the clusters in Figure 2 and Figure 3. *EVA1* is represented by two RefSeqs.

**Figure 5:**
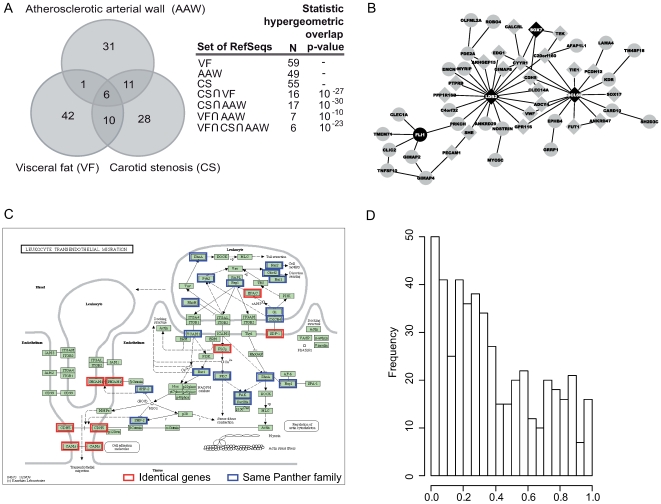
Intersection, network and bioinformatic analyses of the A-module. (A) Venn diagrams showing overlaps of genes in the A-module (three clusters related to extent of atherosclerosis) (Figure 2, Figure 3, Figure 4). Seven genes were found in both the atherosclerotic arterial wall and visceral fat clusters (*P* = 10^−10^), 17 in the atherosclerotic arterial wall and carotid stenosis clusters (*P* = 10^−30^), and 16 in the visceral and carotid stenosis clusters (*P* = 10^−27^). Six genes were found in all three clusters (*P* = 10^−23^). The union of all three clusters represented 128 genes. (B) A gene regulatory network inferred by co-expression of A-module genes using genome-wide expression data from the atherosclerotic arterial wall, carotid stenosis tissue, and visceral fat. Network edges are supported by at least two of the datasets, resulting in a total of 49 nodes. Marked in black are nodes (genes) with known regulatory activity, which are prioritized by the algorithm (Text S1). Marked as diamonds are 24 genes present in intersections between at least two of the clusters in Figure 5A (n = 27). (C) The TEML pathway. Marked in red are eight genes in the A-module that perfectly matched genes in the TEML pathway (*P* = 6.6×10^−5^). Marked in blue are 15 genes in the A-module that were associated with the TEML pathway according to Panther family annotation in DAVID. For a list of all genes in the TEML pathway and Panther families see Table S7 and Table S8, respectively. (D) The *P*-value distribution of 484 eSNPs (SNPs with allele distribution affecting gene expression) in the A-module indicating association with CAD according to a recent GWAS, the WCTTT study [10].

The molecular underpinnings of atherosclerosis are believed to be very similar in all major arteries [7]. Accordingly, if the two atherosclerosis-related tissue clusters identified in the STAGE cohort are of general importance for atherosclerosis, they should be possible to confirm, at least in part, in another atherosclerotic tissue sample. To this end, total RNA samples from atherosclerotic carotid lesions were isolated from patients undergoing carotid stenosis surgery (Figure 1 and Table 1). Both the gene expression profiling and the subsequent two-way clustering analysis were performed exactly according to the protocol used for the STAGE cohort. A well-established surrogate measure of the extent of carotid atherosclerosis [8], the intima-media thickness (IMT), was determined preoperatively using ultrasound. The first clustering step generated a total of eight tissue clusters (Table S1) representing 904 RefSeqs/894 genes. In the second clustering step, one of the eight tissue clusters (n = 55 RefSeqs/54 genes, Table S4, (90% CI: 32–55)) segregated the patients into two groups according to IMT score (*P* = 0.039, Figure 4). Remarkably, 16 of the 55 RefSeqs overlapped with genes in the visceral fat cluster (*P* = 10^−27^), and 17 with genes in the atherosclerotic arterial wall cluster (*P* = 10^−30^) (Figure 5A). Six RefSeqs (representing the genes encoding C-type lectin domain family-14, cadherin-5, chromosome 20 open reading frame-160, endothelial differentiation sphingolipid G-protein-coupled receptor-1, G protein-coupled receptor-116, and LIM domain binding 2 (*LDB2*)) were in all three clusters (*P* = 10^−23^); the union of the clusters contained 129 RefSeqs/128 genes (Figure 5A, Table S5).

### Network and Bioinformatic Analyses of the Atherosclerosis Module

The highly significant overlaps between the three clusters in the atherosclerotic arterial wall, visceral fat and carotid stenosis suggest that the union of all genes may represent a module harboring biological activity important for human atherosclerosis (referred to as the A-module). To investigate interactions between genes in the A-module, gene expression profiles from these tissues were reused to infer a total of three gene networks (Text S1). In Figure 5B, a network supported by nodes and edges in at least two of the three networks is shown. The network of A-module genes consisted of 49 nodes (genes) interacting with a total of 55 edges, of which *LDB2* had 19 edges and *BCL6B* had 14 edges.

To learn more about the functional representation of the A-module, bioinformatic analysis using Gene Ontology (GO) and KEGG pathway was performed (Table S6). Thirty-one of the 128 genes had previously been related to atherosclerosis (Table S9), 40 had no GO annotation, and six participated in regulatory activity (Text S1). Only 39 of the 128 genes had annotation in KEGG pathways. Twenty-three of these 39 genes (∼60%) were associated with the transendothelial migration of leukocyte (TEML) pathway with a statistical significant enrichment score [9] (*P* = 6.6×10^−5^, FDR = 0.01; Figure 5C).

### Enrichment of Genetic Risk for CAD in the Atherosclerosis Module

If gene activity in the A-module is casually important for atherosclerosis development (and not merely reactive marker for the extent of atherosclerosis), functionally associated single nucleotide polymorphisms (SNPs) in the vicinity of the 128 A-module genes should be enriched for CAD risk. In addition, such enrichment would further strengthen our notion that the A-module genes as being important in atherogenesis. To investigate this, we first identified SNPs in the A-module that were significantly associated with gene expression (eSNPs, indicating a functional relation between the SNP allele distribution and gene expression (Text S1)) using two genetics of gene expression (GGE) studies [10]. Next, to test whether the identified eSNPs also were enriched for association with CAD, we assembled results from a recent genome-wide association study (GWAS), the Wellcome Trust Case Control Cohort (WTCCC) study [11]. Since the GGE and WTCCC studies used different SNP-microarray platforms, strong linkage disequilibrium (LD) (R>0.84) was used to confer matches between eSNPs and WTCCC SNPs resulting in a set of 484 eSNPs. The distribution of *P*-values for CAD associations according to the WTCCC study for these 484 eSNPs is shown in Figure 5D. To determine whether this distribution was significantly enriched for CAD risk, we empirically estimated the null distribution of 100,000 random sets of 484 WTCCC eSNPs. 10.3% of the 484 eSNPs in the A-module had a significant association to CAD (*P*<0.05), compared to an average of 5.8% of the eSNPs (95% CI: 2.5%–9.2%) in the random sets (Z = 2.64; *P* = 0.004), representing a 1.8-fold enrichment of CAD risk in the A-module. When instead all SNPs were considered, the enrichment of CAD risk in the A-module was 1.4-fold (Z = 2.71; *P* = 0.003).

### Identifying a Putative Regulator of the Atherosclerosis Module

Of the six genes in the intersection of all three clusters making up the A-module (Figure 5A), *LDB2* was the only transcriptional regulator. The re-occurrence of this transcriptional co-factor in three separate genome-wide analyses suggested a regulatory role of the A-module genes. A notion supported by the interconnectivity of *LDB2* in the network analysis (Figure 5B). To investigate this possibility further, we first identified seven transcription factors (TFs) (ISL-1alpha, Lmo2, Lhx3a, Lhx3b, LHX2, LHX4, and BRCA1) having LIM-binding domains [12] or otherwise previously been shown to interact with LDB2 [13]. We then performed *in silico* sequence matching for 161 promoters (Ensembl) found in 122 of the 128 A-module genes using TRANSFAC (v11.2) [14]. Of these 161 promoters (target promoters), 81% had binding site(s) for at least one of the seven TFs, suggesting that LDB2 could regulate the A-module via these TFs. In relation to a background of 10,255 human promoters covering a [-600,-1] region relative to transcription start sites, binding to the target promoters was enriched 1.2- to 5-fold (Text S1, Table S10). The enrichment for the entire family of 7 TFs was statistically significant (*P* = 0.011).

### Functional Validation of LDB2 in Atherosclerosis

**Figure 6:**
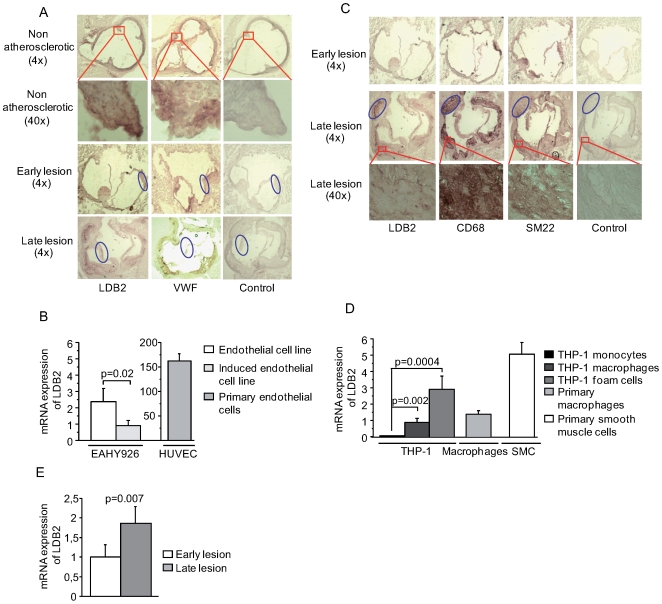
LDB2 expression in atherosclerotic lesions and cultured lesion cell types. Total RNA was isolated from cell cultures and mouse aortic arch (third rib to aortic root). Consecutive mouse aortic root sections were incubated with goat anti-LDB2, rat monoclonal anti-mouse CD68, rabbit polyclonal anti-mouse SM22 alpha, or rabbit polyclonal anti-human VWF at 4°C overnight and counterstained with hematoxylin. RT–PCR was performed on total RNA isolated from human pulmonary artery SMCs, THP-1 monocytes, THP-1 macrophages generated with phorbol 12-myristate 13-acetate, THP-1 foam cells cultured from THP-1 macrophages incubated with acetylated low density lipoproteins, primary macrophages differentiated from primary monocytes isolated from human blood with AB serum, cultured EAHY926 cells, EAHY926 cells induced with 20-ng/ml human recombinant TNF-α, and HUVECs isolated with collagenase. (A) Mouse LDB2 and VWF protein expression in serial sections of aortic roots from *Ldlr* ^−/−^ *Apob* ^100/100^ mice at 10 weeks (arterial wall without visual atherosclerosis, “non-atherosclerotic”), 20 weeks (early lesions, fatty streaks), and 50 weeks (late lesion, plaques). Ovals indicate areas of overlapping LDB2 and VWF staining in relation to negative controls. (B) *LDB2* mRNA levels in EAHY926 cells, induced EAHY926 cells, and HUVECs (*n* = 4 per cell type; scales on Y-axes are comparable because the RT-PCR was performed in one single run). (C) Mouse LDB2, CD68, and SM22 alpha protein expression in serial sections of aortic roots from *Ldlr* ^−/−^ *Apob* ^100/100^ mice at 20 and 50 weeks. (D) *LDB2* mRNA levels in primary human SMCs, THP-1 monocytes, THP-1 monocytes differentiated into THP-1 macrophages, THP-1 foam cells, and primary human monocytes differentiated into macrophages (n = 4 per experiment). Ovals indicate areas of overlap between LDB2 and CD68 but no or very subtle SM22 staining in relation to negative controls. (E) mRNA levels measured by real-time PCR from late (40 weeks, plaques, n = 5) and early (20 weeks, fatty streaks, n = 5; lesions from the aortic arch in *Ldlr* ^−/−^ *Apob* ^100/100^ mice.

Next, we investigated the possible role of LDB2 in atherosclerosis *in vitro* in three major atherosclerosis cell types as well as *in vivo* in atherosclerosis-free arterial wall and in early and late atherosclerotic lesions in atherosclerosis-prone *Ldlr* ^−/−^ *Apob* ^100/100^ mice [15]. The presence of LDB2 in the arterial endothelium was first assessed by co-localization of LDB2 with the endothelial marker von Willebrand factor (VWF). LDB2 expression was most obvious in the endothelium before an atherosclerotic lesion had developed and generally co-localized with VWF (Figure 6A, 40×). In late and early lesions, LDB2 endothelial expression was patchy and subtler, and the co-localization with VWF was less clear except in the endothelium of lesion-free areas (e.g., cusps; Figure 6A). *LDB2* expression in endothelial cells was confirmed by RT-PCR analyses in a human endothelial cell line (EAHY926) and in human umbilical vein endothelial cells (HUVECs) (Figure 6B). In accordance with the immunohistochemical results, the mRNA levels were higher in noninduced than in induced EAHY926 cells (Figure 6B).

To investigate LDB2 protein expression in other atherosclerosis cell types, CD68 was used as a marker of lesion macrophage/foam cells and SM22 (transgelin) as a marker of lesion smooth muscle cells (SMCs). In early lesions, LDB2 staining was subtle (but clearly present compared to control) and appeared to co-localize with both CD68 and SM22 (Figure 6C). In late lesions, LDB2 staining was marked, and in all locations of LDB2 staining there was also CD68 staining. In this sense, there was co-localization of LDB2 and CD68. However, the CD68 staining was generally stronger, and some areas with CD68 staining had little or no LDB2 staining. LDB2 also co-localized with SM22, but some areas with marked LDB2 staining had no SM22 staining (Figure 6B, ovals). LDB2 was also expressed in macrophages/foam cells in human carotid lesions (Figure S2).

The immunohistochemical results were largely confirmed by RT-PCR analyses of primary SMCs and macrophages and a human monocytic cell line (THP-1) (Figure 6D). Consistent with the higher protein expression in late lesions than in early lesions, *LDB2* mRNA levels increased with differentiation of THP-1 monocytes to macrophages and foam cells (panel 1). The expression of *LDB2* in THP-1 was also confirmed in primary macrophages (panel 2). In primary SMCs isolated from human pulmonary artery, there was also clear expression of *LDB2*, which in comparison with the immunohistochemical results was surprisingly high (panel 3).

In summary, LDB2 was expressed by all three major atherosclerosis cell types; before lesion formation and in early lesions primarily in the endothelium and in late lesions, mainly in macrophages/foam cells but also in SMCs. The generally higher LDB2 expression in late lesions was confirmed by RT-PCR of total RNA from early and late lesions isolated from mouse aortic arch samples (Figure 6E).

Last, we examined mRNA levels of 20 genes central to TEML in the arterial wall of 6-week-old *Ldb2* ^−/−^ mice. Our goal was to investigate a possible role of LDB2 as a regulator of TEML genes in general and specifically as a regulator of A-module genes. All 20 genes had higher levels of expression in *Ldb2* ^−/−^ than in wild-type mice whereof 13 was significantly higher (Table 2). Eight of these 13 genes were specific to the A-module, and five were not. Of note, five of the investigated genes have previously been targeted in mouse models of atherosclerosis and found to be affecting lesion development [16]–[20].

**Table 2:** mRNA levels measured by real-time PCR from the aortic arch of 6-week-old mice deficient in Ldb2 (Ldb2^−/−^) and littermate wild-type controls (Ldb2^wt/wt^).

Category	Gene Symbol	Ldb2^wt/wt^	Ldb2^−/−^	*p*-Value
**A-module genes associated to TEML**				
Claudin 5	Cldn5	307±108	397±271	0.47
Phospholipase C gamma 2	Plcg2	461±65	726±219	0.019
Cadherin 5	Cdh5	352±114	603±179	0.011
Chemokine (C-X-C motif) ligand 12	Cxcl12	498±103	715±168	0.015
Platelat/endothelial cell adhesion molecule	Pecam1	345±122	564±157	0.016
Angiotensin II receptor-like 1	Aplnr	435±253	846±404	0.069
Kinase insert domain receptor	Kdr	386±224	964±555	0.043
Protocadherin 12	Pcdh12	491±188	785±339	0.10
Protein Kinase N3	Pkn3	410±193	1076±697	0.050
Protein kinase C eta	Prkch	547±199	1045±369	0.019
Protein tyrosine phosphatase receptor type B	Ptprb	486±167	1115±575	0.030
Tek tyrosine kinase (endothelial)	Tek	430±122	1068±551	0.021
Tyrosine kinase with immunoglobulin-like and EGF-like domains 1	Tie1	524±170	895±374	0.056
**Other TEML genes**				
Intercellular adhesion molecule 1	Icam1	405±54	533±73	0.0042
F11 receptor	F11r	388±59	614±151	0.0037
Junction adhesion molecule 2	Jam2	452±70	616±137	0.018
Junction adhesion molecule 3	Jam3	567±53	741±163	0.022
Vascular cell adhesion molecule 1	Vcam1	492±83	730±134	0.0025
Thymus cell antigen 1	Thy1	556±158	707±264	0.23
CDC42 effector protein (Rho GTPase binding) 5	Cdc42ep5	540±127	622±119	0.26

Values are mean ± SD. *p*-Values are calculated with unpaired t-test.

Values are normalized to acidic ribosomal phosphoprotien P0 and TATA box binding protein.

*Ldb2* ^−/−^, n = 5–6; *Ldb2* ^wt/wt^, n = 6–7.

Taken together, the functional validation supports a role for LDB2 in TEML and atherosclerosis development. Particularly, since endothelial LDB2 seems to regulate TEML already before microscopic evidence of lesion formation.

## Discussion

In the STAGE study, we profiled five CAD-relevant tissues to identify functionally associated genes with potential importance in coronary atherosclerosis. This analysis revealed 128 genes that were strongly associated with atherosclerosis severity (A-module). The A-module was found to be enriched with genetic risk for CAD and involve the TEML pathway. Parts of the A-module were active in both atherosclerotic arterial wall and visceral fat. The latter may be a local source of inflammation contributing to coronary atherosclerosis. We also identified a putative high-hierarchy regulator of the A-module, LDB2, which was robustly expressed in all major lesion cell types both in lesion-free and in late atherosclerosis lesions. Interestingly, key genes in the TEML pathway were differentially regulated in the arterial wall of *Ldb2*-deficient mice. Our findings suggest that the A-module, including LDB2, is important in the regulation of TEML and in atherosclerosis development.

TEML is an established pathway in atherosclerosis and other inflammatory diseases [21]. Transendothelial migration of monocytes is essential for foam-cell formation and for early phases of atherogenesis, and transendothelial migration of T-cells may be central in later phases [22]. Indeed, leukocyte migration has been suggested as a therapeutic target [23]. The identified module was enriched in genes involved in TEML and thus may be causally involved in the development of clinically significant atherosclerotic lesions (as indicated by the extent of coronary stenosis and IMT). However, most of the identified A-module genes lack pathway annotations but may in future studies be proven important to leukocyte migration or its regulation.

The STAGE study was designed as a “top-down” systems biological approach to identify gene networks or groups of otherwise functionally associated genes (modules) of importance for disease severity [3]. The term “top-down” refers to our belief that these modules must first be identified in clinical studies as the most disease relevant and then be consecutively detailed by studies in animal and cellular models to reveal high-resolution networks [24]. In contrast, “bottom-up” systems biology approaches first identify full biological networks in prokaryotic or yeast cells and then examine their roles in more disease-relevant systems. Systems biological approaches have advantages over traditional gene-expression profiling studies, which usually focus on identifying individual genes differentially expressed as a result of disease. Such gene-by-gene analyses generate many false positives due to a vast “multiple testing” problem. In contrast, the two-way clustering approach first focuses on identifying functionally associated genes (which in the current study reduced the number of genes from 12,621 to 3958 represented in 60 tissue clusters) and then investigate whether the generated clusters (not individual genes) are related to a given disease phenotype.

Using a multi-organ approach [3], we hypothesized the liver, skeletal muscle, or fat deposits would harbour functionally related genes (e.g., clusters, modules, networks) reflecting molecular processes in those organs affecting the levels of inflammatory mediators, blood lipids, glucose or unknown blood constituents that contribute to coronary atherosclerosis development. There were no clusters relating to the extent of coronary atherosclerosis in the liver and skeletal muscle. This was surprising given the importance of these organs for CAD risk factors, such as plasma cholesterol and diabetes. However, therapies to reduce plasma lipid and glucose levels (Table 1) might have normalized disease-promoting activities in CAD-modules in these organs. In contrast, we identified one part of the A-module in visceral fat that segregated patients according to the degree of coronary stenosis. Although the relation of visceral fat to CAD risk factors in blood is less clear, a high waist-hip ratio—an indicator of increased visceral fat mass in the abdomen—is a strong predictor of CAD [25]. An interesting aspect of the visceral fat in the mediastinum is its anatomic location and the possibility that it is a source of local macrophages releasing inflammatory mediators [26]. Another possible cellular source for the presence of the TEML-enriched atherosclerosis module in visceral fat may be endothelial cells, which are relatively enriched in this tissue. Although our study does not directly address the subcellular origin of the A-module in visceral fat or how it contributes to atherosclerosis, it might be a local source of inflammatory mediators that increase the rate of atherosclerosis progression [27].

In all, 60 tissue clusters were identified, two of which—one in atherosclerotic lesion and one in visceral fat—related to the extent of coronary atherosclerosis. This might appear to be a small fraction (2/60, ∼3%). However, since the first clustering step takes no phenotypic data into consideration but is entirely based on the mRNA signals in each tissue, these 60 clusters may relate to tissue physiology or subtraits of CABG patients (Table 1). Examining the latter possibility, we found that as many as 41 of the tissue clusters (besides the two related to extent of coronary atherosclerosis) segregated the patients into groups with significant difference in the levels of subtraits (not shown).

The gene expression clustering was done with the absolute value of Spearman rank correlation as distance measure. Thus, we also included inverse correlated genes which could be implicated in the same pathway and functionally related. Moreover, Spearman rank correlation is a non-parametric measure stable against outliers and in this sense a better distance measure than commonly used Euclidean and Manhattan distances, where the magnitude in expression levels are important. Of note, a clustering algorithm could produce different clusters depending on the distance measure used and the A-module could therefore have been different or even lost by other metric clustering choices.

We used atherosclerotic aortic wall/internal mammary artery (IMA) ratios to highlight atherosclerosis gene expression in the aortic wall because both aortic wall and IMA samples contain normal wall gene expression. Unlike the aortic wall, however, the IMA has no atherosclerosis [28]. This notion was supported by macro- and microscopic examinations of randomly chosen sets of aortic wall and IMA samples. Moreover, two-way clustering of mRNA signals from the aortic wall samples alone did not generate any cluster that segregated patients by stenosis scores (not shown), which may be due to a relative large portion of normal vascular wall gene expression in this tissue. However, we cannot entirely exclude the possibility that using the aortic wall/IMA ratios resulted in some false-positive genes (nonatherosclerosis genes related to normal vascular wall gene expression) that should have been excluded from the A-module or false-negative genes that otherwise should have been included.

We decided to use two different atherosclerosis cohorts—coronary for the exploration and carotid for the confirmatory step. In doing so, we added more credibility to the confirmatory step that would have been lost if we instead had used identical cohort for exploration and confirmation. The validation in the carotid cohort indicates a general importance of the A-module in atherosclerosis and at the same time rules out the possible risk that any of the tissue clusters identified in the STAGE cohort was a result of the exploratory study design (e.g. choice of sample locations and/or using ratios instead of straight expression) rather than related to atherosclerosis. The extents of coronary and carotid atherosclerosis (as judge from the surrogate measurements of stenosis score and IMT [8],[29]) have repeatedly been shown to be highly correlated [30]. This observation is not entirely surprising since atherosclerosis development and the principal molecular processes underlying this development have been found to be very similar in all major arteries, regardless of location [7].

Currently, GWAS are given much attention in leading scientific journals. However, such studies have some limitations, since they are primarily designed to identify the relatively few DNA variants that influence the risk of developing complex diseases, like CAD, independently of other risk factors [31]. In the current study, we used a recently published GWAS [11] to further validate the A-module genes by calculating the relative enrichment of genetic CAD risk in the module. Unlike today's GWAS, which link DNA variation directly to clinical phenotypes, future studies that also include intermediate expression phenotypes have the potential to extract much more disease-relevant information on DNA variation that contributes to the development of complex diseases. For now, this information remains hidden in the data generated by GWAS.

Genes encoding LIM domain-binding factors such as *LDB2* were initially isolated in a screen for proteins that physically interact with the LIM domains of nuclear proteins. These proteins bind to a variety of TFs and are likely to function as enhancers, bringing together diverse TFs to form higher-order activation complexes [32]–[33]. Our screen of LDB2-associated TFs identified ISL-1alpha, Lmo2, Lhx3a, Lhx3b, LHX2, LHX4, and BRCA1. ISL-1alpha enhances HNF4 activity and thus insulin signaling [34]–[35]. Lmo2 is involved in angiogenesis [36]–[37]. Lhx3 and Lhx4 regulate proliferation and differentiation of pituitary-specific cell lineages [38] and are expressed in subsets of lymphocytes [39] and thymocyte tumor cell lines [40]. BRCA1 is associated with a selective deficiency in spontaneous and LPS-induced production of tumor necrosis factor (TNF)-α and of TNF-alpha-induced expression of intercellular adhesion molecule-1 (*ICAM1*) on peripheral blood monocytes [41] and in controlling the life cycle of T-lymphocytes [42]. LDB2 has not previously been related to CAD or atherosclerosis. Because of its high-hierarchy regulatory role and involvement in diverse biological processes, LDB2 is an interesting target for further evaluation in complex diseases.

Being the only transcriptional regulator among the six genes relating to severity of atherosclerosis present in all three tissue clusters (Figure 6A), LDB2 was chosen for functional validation in atherosclerosis. However, despite the fact that none of the other five genes were transcriptional regulators, they might still be of functional importance for atherosclerosis development, which remains to be determined. In nonatherosclerotic arterial wall and in early lesions, LDB2 was mainly expressed by the endothelium. In late lesions, LDB2 expression was more intense and mainly seen in macrophages/foam cells but also in SMCs. The TEML pathway has been implicated in both early and late atherosclerosis [23]. This pathway is also active in lesion SMCs accompanying endothelial cells in recruiting monocytes from the blood to the atherosclerotic plaque [43]–[44]. The pattern of *LDB2* expression seen in early and late lesions has been observed for other key TEML genes (*Vcam1*, *Icam1*, *Cxcl1*, -*14*, and -*16*, and *Cdc*20) [45]. The notion that LDB2 is an important regulator of TEML is further supported by the fact that 13 key genes in TEML were differentially expressed in the arterial wall of *Ldb2* ^−/−^ mice already at 6 weeks of age. Five of those genes have previously been shown to affect atherosclerosis in mouse model studies [16]–[20]. In addition, a very recent study demonstrated that LDB2 regulates cell migration both *in vitro* and *in vivo* [46]. However, the final verdict on LDB2 as an important regulator of atherosclerosis development remains to be determined.

Although it cannot be excluded that the A-module also will be of importance for early stage of atherosclerosis (e.g., by promoting early lesion development through activating TEML in the atherosclerosis-free endothelium), the current study mainly supports a role of the A-module in late stages of coronary atherosclerosis. If the activity of this cassette of genes is mirrored, at least in part, by gene expression in blood (i.e., in leukocytes) or by plasma protein levels, the A-module may be helpful as a complement to semi-invasive investigations (e.g., angiography) as markers of degree of coronary and carotid stenosis.

In conclusion, by adopting a new strategy for functional analysis of expression profiles isolated from multiple CAD-relevant organs, we identified a module that is genetically enriched with CAD risk and important for TEML and atherosclerosis development. The clinical usefulness, and exact role in CAD of this module and its high-hierarchy regulator [32]–[33] LDB2, merit further investigation.

## Methods

### Study Patients, Biopsy Collection, and Follow-Up

The STAGE study enrolled 124 patients undergoing CABG at Karolinska University Hospital, Solna. Forty-two patients undergoing carotid surgery at Stockholm Söder Hospital were recruited as a confirmatory cohort. The studies were approved by the Ethics Committee of Karolinska University Hospital. All patients gave written informed consent.

Tissue samples from the distal IMA, wall of the ascending aorta (aortic root) at the site of proximal vein anastomosis, anterior hepatic edge (liver), skeletal muscle, and visceral fat in the mediastinum were preserved in RNAlater (Qiagen) and frozen at −80°C. Lesions in aortic wall samples [47]–[48] and the absence of lesions in the IMA [28] were confirmed by macroscopic and microscopic examinations (not shown). Carotid plaques were embedded in OCT (Histolab Products), frozen in liquid isopentane and dry ice, and stored at −80°C.

One hundred fourteen CABG and 39 carotid stenosis patients came to a 3-month follow-up visit. Using a standard questionnaire, a research nurse obtained a medical history and lifestyle information (e.g., smoking, alcohol consumption, and physical activity). A physical examination was performed including venous blood sampling (Text S1).

### Coronary and Carotid Atherosclerosis Measurements

All CABG patients underwent preoperative biplane coronary angiography (Judkins technique). Angiograms were evaluated with QCA techniques (Medis). The left and right coronary arteries and their branches were divided into segments [49]. Each segment was measured during end-diastole. A stenosis score was calculated from all major lesions in the coronary arteries (1 point, 20–50% luminal obstruction; 2 points >50% obstruction). In some patients, right coronary artery occlusion prohibited QCA evaluation. Before surgery, carotid arteries were examined with B-mode ultrasound. The far wall of the common carotid artery was used to measure IMT from the endarterectomy side [50].

### RNA Isolation and Expression Profiling

We performed gene expression profiling on three tissues (liver, skeletal muscle, visceral fat) in 66 of 114 STAGE patients, and also in 40 of these 66 patients, on atherosclerotic arterial wall and IMA. In the validation cohort, 25 carotid lesions from 39 patients were randomly selected for RNA isolation and gene expression profiling. Aortic arches (third rib to aortic root) were isolated in RNA later (Ambion) from 6-week-old mice deficient in *Ldb2* (*Ldb2* ^−/−^; Mutant Mouse Regional Resource Center, University of California, Davis), heterozygous and wildtype littermates, and 20- and 40-week-old atherosclerosis-prone mice deficient in the low density lipoprotein receptor and expressing exclusively apolipoprotein B100 (*Ldlr* ^−/−^ *Apob* ^100/100^ mice). Total RNA was isolated from all biopsies with Trizol (BRL-Life Technologies) and FastPrep (MP Biomedicals) and purified with RNeasy Mini kit using DNase1 treatment (Qiagen). Sample quality was assessed with an Agilent Bioanalyzer 2100. cRNA yield was assessed with a spectrophotometer (ND-1000, NanoDrop Technologies) before hybridization to HG-U133 Plus 2.0 arrays (Affymetrix). The arrays were processed with a Fluidics Station 450, scanned with a GeneArray Scanner 3000, and analyzed with GeneChip Operational Software 2.0.

### Immunohistochemistry

Mouse aortic roots (aortic valve level) and human carotid lesions were isolated and frozen in liquid nitrogen, embedded in OCT compound (Histolab Products), cryosectioned (5 µm), and fixed in acetone. Endogenous peroxidase activity was quenched with 0.3% hydrogen peroxide/0.01% NaN_3_ in water for 10 minutes, and sections were incubated with 5% blocking serum. Consecutive sections were incubated with goat anti-LDB2 (Santa Cruz Biotechnology) [51], rat monoclonal anti-mouse CD68 (Serotec), mouse monoclonal anti-human CD68 (Novocastra Laboratories), rabbit polyclonal anti-mouse SM22 alpha (transgelin, Abcam), or rabbit polyclonal anti-human VWF (DakoCytomation) at 4°C overnight. In negative controls, primary antibody was replaced with serum. After rinsing in Tris-buffered saline, sections were incubated with secondary biotinylated bovine anti-goat, anti-mouse, or anti-rat (Vector Laboratories) or anti-rabbit IgG (DakoCytomation). Avidin-biotin peroxidase complexes (Vectastain ABC Elite, Vector Laboratories) were added followed by visualization with DAB (Vector Laboratories). All sections were counterstained with Gill hematoxylin (Histolab Products).

### Cell Cultures

THP-1 monocytes were plated in 10% fetal calf serum/RPMI-1640 with L-glutamine (2 mM) and HEPES buffer (25 mM) (Gibco-Invitrogen) supplemented with penicillin (100 U/ml) and streptomycin (100 µg/ml) and differentiated into macrophages with phorbol 12-myristate 13-acetate (50 ng/ml) (Sigma) for 72 hours. To generate foam cells, macrophages were incubated with acetylated low density lipoproteins (50 µg/ml) for 48 hours. Human monocytes were isolated from blood with Ficoll/Hypaque as described [52], placed in six-well dishes, and allowed to adhere overnight in RPMI-1640 supplemented with penicillin (100 U/ml), streptomycin (100 µg/ml), and 10% pooled human AB serum. After washing, fresh serum-containing medium was added, and cells were cultured for 6 days and harvested. EAHY926 cells were cultured in DMEM containing high glucose, penicillin (100 U/ml), streptomycin (100 µg/ml), 10% fetal calf serum, hypoxanthine (100 µmol/l), aminopterin (0.4 mmol/l), and thymidine (16 mmol/l). HUVECs were obtained by collagenase treatment, cultivated, and identified as described [53]. SMCs from human pulmonary artery (Clonetics) were cultured in SmGm2 medium containing growth factors (Clonetics) as described [54].

### Real-Time PCR

Total RNA (0.15 µg) was reverse transcribed with Superscript III (Invitrogen). After threefold dilution, cDNA (3 µl) was amplified by real-time PCR with 1xTaqMan universal PCR master mix (Applied Biosystems) on an ABI Prism 7000 (PE Biosystems) using Assay-On-Demand kits containing corresponding primers and probes (Applied Biosystems). mRNA levels were normalized to acidic ribosomal phosphoprotein P0 and TATA-box binding protein. Samples were analyzed in duplicate.

### Pre-Processing of Gene Expression Data

Gene-expression values were pre-processed with the robust multichip average [55] procedure in three steps (background adjustment, quantile normalization, summarization). Of 604,258 perfect-match Affymetrix probe signals, 423,636 were mapped to transcripts using RefSeq numbers as identifiers [56], generating 15,042 RefSeq transcripts corresponding to 12,621 genes. Straight expression values (i.e., mRNA signals obtained from one microarray) were used for data analyses of all tissue biopsies (including the carotid lesion biopsy in the confirmatory cohort) except for the atherosclerotic arterial wall and IMA. The latter two biopsies were combined in atherosclerotic arterial wall/IMA mRNA ratios before data analysis. mRNA signals in the atherosclerotic arterial wall biopsy reflect gene activity in the atherosclerotic lesion and in normal arterial wall, whereas mRNA signals in the IMA mainly reflect normal arterial wall gene activity (the IMA is almost entirely devoid of atherosclerotic lesions) [28]. Thus, the use of atherosclerotic arterial wall/IMA ratios highlights gene activity related to atherosclerotic lesions in arterial wall and excludes that relating to normal arterial wall.

### Two-Way Clustering

Coupled two-way clustering [4]–[6] was performed to identify small and stable clusters of related signals of importance for CAD. In the first step, clusters were defined using superparamagnetic clustering [4], with the absolute value of Spearman rank correlation as a distance measure between genes. Spearman rank is a non-parametric measure which is robust to outliers and by using absolute values we also put together anti-correlated genes. The analysis was done without using any predefined conceptions (i.e., phenotypes of the patients). Genes that did not belong to a cluster were excluded. Then, in the second step, the identified clusters were related to coronary atherosclerosis by hierarchical clustering [57] of the patients, using Manhattan distance and average linkage as distance measures, based on the mRNA signals in each of the clusters defined in the first step (Text S1).

To assess the repeatability and reliability of these clusters, resampling using Jackknife analysis was performed [58] (Text S1).

### Genetic Enrichment Analysis

A-module genes were mapped to eSNPs (Text S1) using two GGE studies [10] and tested for enrichment of association with CAD using the results from the WTCCC study [11]. Different SNP panels were used in the GGE and WTCCC studies, therefore we included eSNPs and all SNPs in strong LD (R>0.84) with the eSNPs. In the 128 A-module genes, there were 97 eSNPs and 387 LD SNPs of the eSNPs, resulting in an expanded set of 484 eSNPs. Random sampling strategy was used to assess whether the expanded eSNP set was more likely to associate with CAD than randomly selected sets of SNPs of equal number. In each random sample, 97 SNPs located within 1 megabase of human gene regions were selected to ensure the location of the random SNP sets matched that of the eSNP set in the A-module. The randomly selected SNP sets were then expanded by including SNPs in strong LD (*R*>0.84) with any of the randomly identified SNPs. We required the final size of the expanded random set of SNPs to be within ±10% of the expanded set of eSNPs in the A-module. Therefore, the random sampling scheme produced sets of SNPs in which the LD, set size, and location with respect to protein coding genes matched those of the expanded eSNP sets in the A-module. The process was repeated 100,000 times. For each random SNP set, we counted the percentage of SNPs with association *P*-value to CAD<0.05, and constructed the null distribution. The enrichment *P*-value was calculated as the number of times that the percentage exceeds 10.3% from random sampling divided by 100,000.

### Statistical Analysis

Clinical and metabolic characteristics are given as continuous variables with means ± SD and as categorical variables with percentages and numbers of subjects. *P*-values were calculated with unpaired *t* tests; skewed values were log-transformed. Statistical significances in Venn diagrams were computed using hypergeometric distributions (Text S1). GO and pathway analyses were performed with DAVID (Database for Annotation, Visualization and Integration Discovery) software [9]. Mathematica 5.2 or StatView 5.0.1 was used for all other calculations. Text mining was used to define transcripts previously related to CAD and atherosclerosis (Text S1, Table S9). For promoter analysis, TRANSFAC (v11.2) [14] was used (Text S1).

## Supporting Information

Figure S1 Principles of the cost function in the SPC algorithm. The superparamagnetic clustering (SPC) algorithm uses a cost function with a temperature parameter (T) to assign genes into different clusters. Genes could belong to many clusters (right) or to no cluster at all (left). At a certain temperature the clusters are robust and stable against noise (middle).
(1.21 MB EPS)

Figure S2 LDB2 proteins and CD68 staining in serial sections of human carotid plaques. Consecutive human carotid plaque sections were incubated with goat anti-LDB2 antibody and rat monoclonal anti-mouse CD68 at 4°C overnight. LDB2 is co-localized with CD68.
(3.17 MB EPS)

Table S1 Gene expression cluster relation to surrogate measurements of atherosclerosis (QCA and IMT).
(0.04 MB XLS)

Table S2 49 RefSeqs corresponding to 48 genes of the atherosclerotic arterial wall/IMA cluster in Figure 2.
(0.02 MB XLS)

Table S3 59 RefSeqs/genes of the visceral fat cluster in Figure 3.
(0.03 MB XLS)

Table S4 55 RefSeqs corresponding to 54 genes of the carotid lesion cluster in Figure 4.
(0.02 MB XLS)

Table S5 129 RefSeqs corresponding to 128 genes in the A-module.
(0.04 MB XLS)

Table S6 GO and pathway analysis of the three clusters and the union of all three clusters.
(0.03 MB XLS)

Table S7 TEML pathway genes in DAVID (n = 117).
(0.03 MB XLS)

Table S8 Panther family classification of genes in TEML and the atherosclerosis module (http://www.pantherdb.org/).
(0.03 MB XLS)

Table S9 2,832 genes previously associated to CAD.
(0.38 MB XLS)

Table S10 Binding sites of transcription factors related to LDB2 among the upstream sequences of the 128 genes in Table S5 as compared to a background set of sequences.
(0.04 MB XLS)

Text S1 Supporting methods.
(0.04 MB PDF)
