# Supplementary material for: Multi-Organ Expression Profiling Uncovers a Gene Module in Coronary Artery Disease Involving Transendothelial Migration of Leukocytes and LIM Domain Binding 2: The Stockholm Atherosclerosis Gene Expression (STAGE) Study
Source: PLoS Genet. 2009 Dec 4;5(12):e1000754. doi: 10.1371/journal.pgen.1000754 (PMC2780352; doi:10.1371/journal.pgen.1000754)
Supplement: Text S1 — Supporting methods. (0.04 MB PDF) [file pgen.1000754.s013.pdf]

Supporting Methods

Study Patients and Tissue Sample Collection

One hundred twenty-four patients who underwent elective coronary artery bypass grafting (CABG) at the Karolinska University Hospital, Solna from 2002 to 2005 were included in the STAGE study. Exclusion criteria were severe diseases (e.g., cancer, kidney disease, and chronic systemic inflammatory diseases) other than coronary artery disease (CAD). The number of surgeons collecting tissue samples during the CABG was restricted to four. A strict protocol was used to standardize the collection procedure, including a specific order for obtaining each tissue sample in relation to the start of surgery. Anesthesia was standardized to keep systolic blood pressure <150 mm Hg throughout the operation. The time of extraction of each biopsy, deviations from the protocol, and no routine events were noted.

A total of five tissue samples were obtained. Skeletal muscle (~0.5 g) was taken from the medial border of the apical rectus abdominus muscle close to the incision in sternum. Visceral fat (~1 g) was taken from the mediastinal tissue anterior to the pericardium and great vessels. The internal mammary artery (IMA) was dissected from the inside of the left chest wall, and 1 cm of the distal part of the artery was collected. Aortic wall samples were obtained from the whole punch used to create the proximal vein graft anastomoses at the aortic root during the operation. Liver tissue (3 mm diameter, 0.05 g) was taken from the very inferior border of the left liver lobe at the end of the operation. This part of the liver was easily accessed after the peritoneum was opened through a small incision in the diaphragm a few centimeters below the xiphoid process. The incisions in the liver and diaphragm were sutured after removal of the biopsy. All tissue samples were taken without use of cautery, and none of the patients suffered from bleeding at the biopsy sites or other complications related to the tissue sampling. The tissue samples were rinsed with RNAlater (Qiagen) to remove blood and immediately (~10 seconds) put into vials

containing fresh RNAlater. The vials were stored at room temperature until the end of surgery, at 4°C overnight, and then frozen at -80°C until further processed.

Laboratory Measurements

Venous blood samples were drawn into precooled sterile tubes (Vacutainer, Becton Dickinson) containing NaEDTA and placed on ice. Plasma was recovered within 30 min by centrifugation (2750 g, 20 min, 4°C) for analysis of cholesterol, triglyceride, and lipoproteins as described (Carlson K. Lipoprotein fractionation. *J Clin Pathol Suppl (Assoc Clin Pathol).* 1973;5:32-37.). Blood glucose was measured by a glucose oxidase method (Kodak Ektachem) and insulin and pro-insulin by enzyme-linked immunosorbent assay (Dako Diagnostics).

The IMA and Non-Atherosclerosis-Related Genes

For reasons unknown, the IMA is an atherosclerosis-free artery (reference 29 in the manuscript). Therefore, we decided to use IMA as an internal control (i.e., from the same patient) to normalize atherosclerotic arterial wall expression related to nonatherosclerotic processes (i.e., normal arterial wall gene expression). This was achieved by using the ratios of atherosclerotic arterial wall/IMA gene chip signals for each RefSeq in the cluster analysis.

However, we wanted to investigate the number of genes involved in “normal nonatherosclerosis-related activity” that differed between the aorta and IMA samples. This was done by analyzing, on a human Affymetrix GeneChip (HG-U133 Plus 2.0), total RNA from an additional human aortic root sample (a pooled aortic RNA sample from three male/female Caucasians who suffered sudden death at an early age [27-45 years]) and thus presumably had little or no aortic atherosclerosis; obtained from Clontech). We found 1.9% of the 15,042 RefSeqs or 285 RefSeqs were differentially expressed (i.e., mRNA levels, FDR=0.05) between this

“normal” aorta and our IMA samples (n=40). None of the 285 RefSeqs were part of the atherosclerosis module (n=129 RefSeqs representing 128 genes).

Clustering Analysis

The principles of the clustering analysis are shown in Figure 1 in the manuscript. Gene-expression data from each tissue were clustered with a coupled two-way approach originally proposed by Getz et al. (reference 5 in the manuscript). The underlying rational for using a two-way clustering approach is mainly to enable clinical phenotypes to groups of functional associated genes rather than individual genes. A consequence of this approach is that all genes not belonging to a cluster (i.e. a putative functional group) will be excluded and not considered for the association analysis with clinical phenotypes (in our case coronary stenosis and IMT). The first step clustering in our analysis of the 278 genome expression profiles reduced the number of RefSeqs to ~4000 from the original ~15000 on the gene chip. The first step of this procedure involves clustering the mRNA signal values on the GeneChip using a superparamagnetic clustering (SPC) algorithm introduced by Blatt et al. (reference 4 in the manuscript) and implemented by Tetko et al. (reference 6 in the manuscript). Unlike hierarchical clustering, this algorithm allows genes to belong to multiple clusters or to no cluster at all. We used SPC because it requires no filtering, is stabile against noise, and selects the optimal number of clusters. It also incorporates a cost function, which penalizes an assignment for placing genes in different clusters. On the other hand, if the distance between two gene-expression profiles is long, then the penalty for putting them into different clusters will be low. The distance *d* between two gene expression profiles e_i and e_j is computed as

$$d_{ij} = 1 - AbsoluteValue\left[SpearmanRankCorrelation\left[e_i, e_j\right]\right].$$

Using a correlation as measure as opposed to a regular distance measure (e.g. Manhattan of Euclidean distance) will prioritize genes with similar expression profiles as opposed to genes with

similar expression levels. Moreover, Spearman rank correlation is non-parametric and thus more robust to extreme (outlier) data points (as compared to Pearson correlation). We use the absolute value of the Spearman rank correlation to accommodate for gene pairs with negative correlation. The cost function uses a parameter called temperature T, whose value varies depending on how the algorithm assigns genes to clusters. At low temperature, all genes will belong to the same cluster; many genes have similar properties, and the system is in an ordered ferromagnetic state. At high temperature, the gene properties change, and the state become unordered and paramagnetic; there will be many clusters (Figure S1). At some value of T, the so-called superparamagnetic state, the system contains some genes in the ordered ferromagnetic state and some in the unordered paramagnetic state that then defines which genes that belongs, or do not belong, to a given cluster (hence, which genes are excluded). The identified clusters were stable over a temperature interval of at least 0.015, and we joined overlapping clusters if they were more than 60% identical and discarded clusters with more than 1000 members. Based on the mRNA GeneChip signal within individual gene clusters generated in the first step, the patients segregated into two groups. In the second step, hierarchical (agglomerative) clustering with average linkage in Mathematica was used. Manhattan distance was used to measure similarities between patients across GeneChip signal values of genes in the cluster. Small patient groups (3 patients or less) were considered outliers and therefore removed; the remaining patients were in such cases re-clustered. To visualize the results of the second clustering step, software introduced by Eisen et al. (reference 56 in the manuscript) was used together with TreeView (reference 56 in the manuscript).

Resampling Analysis

To assess the repeatability and reliability of the clusters, resampling using Jackknife analysis was performed (reference 57 in the manuscript). One patient at the time was removed in the leave-

one-out cross-validation step where after data was reclustered with the SPC algorithm and gene clusters were identified over a temperature. A one-sided 90% confidence interval (CI) was calculated for each cluster (Table S1) showing the reappearance interval from the least number of genes (X) to infinity (i.e. the total number of genes in a given cluster). For example, the visceral fat cluster (n=59 RefSeqs) had a CI starting at 38 to a maximum of 59 RefSeqs. This means that at least 38 RefSeqs of the total 59 in this cluster appear in 90% of the resampling times. Since we are only interested in the lower limit, we performed a one-sided test; infinity in this case is the same as the total number of RefSeqs in a cluster.

Text Mining to Define Genes Previously Associated with CAD and Atherosclerosis

Automated text mining of abstracts in PubMed was used to establish a comprehensive list of genes previously related to CAD and atherosclerosis. Briefly, a gene was considered related if it co-occurred in the abstract text of a published article on PubMed with the MeSH terms *coronary arteriosclerosis*, *arteriosclerosis*, or *atherosclerosis* or the text words *coronary artery disease*, *arteriosclerosis*, or *atherosclerosis*. Gene symbols were extracted and compared to any gene symbol as provided by Entrez. Synonyms were converted to the most commonly used symbol and abbreviations that could be mistaken for other terms were removed. Additional genes were manually extracted from recent reviews of CAD (n=459 genes). The entire list of 2832 CAD- and atherosclerosis-related genes is found in Table S9.

Expression SNPs from GGE

In the GGE (reference 9 in the manuscript), all expression traits were tested for association with

each of the genotyped SNPs. The strongest putative *cis* eQTL for a given expression trait was defined as the SNP most strongly associated with the expression trait over all of the SNPs typed within 1 megabase (Mb) of the transcription start or stop of the corresponding structural gene. The association *p*-values were adjusted to control for testing of multiple SNPs and expression traits using an empirically determined false discovery rate (FDR) constrained to be < 10%. In the case of *trans* eQTL, all SNPs were tested for association to each of the expression traits.

Expression Network Analysis

Three expression networks of the 128 A-module genes were reconstructed from the gene expression profiles of the visceral fat, atherosclerotic arterial wall and carotid stenosis tissues, respectively using ARACNE (Margolin AA et al, ARACNE: an algorithm for the reconstruction of gene regulatory networks in a mammalian cellular context. *BMC Bioinformatics*. 2006 Mar 20;7 Suppl 1:S7.). ARACNE is specifically designed to scale up to the complexity of regulatory networks in mammals using microarray expression profiles. The method uses an information theoretic approach to eliminate the majority of indirect interactions inferred by co-expression methods. From the A-module, genes involved in transcription activities were identified using GO categories (transcription activator activity, transcription coactivator activity, transcription factor binding, transcription repressor activity, transcription regulator activity, transcription cofactor activity, transcription corepressor activity) yielding a list of six genes (*NR1H3, SOX7, FLI1, LDB2, BCL6B, BATF3*). The three networks were overlapped and the final network (Figure 5B) represents the 49 nodes connected with edges supported by at least two of the three networks. The final network is visualized using Cytoscape (Cline MS et al, Integration of biological networks and gene expression data using Cytoscape. *Nature Protocols* 2, 2366 - 2382 (2007)).

No other network or functional analysis of different clusters, other than the three related to

atherosclerosis severity, were tested.

In Silico Promoter Binding Analysis

One hundred sixty-one promoter sequences from Ensembl v. 43 (Hubbard et al, Ensembl 2007, *Nucleic Acids Res*. 2007 Jan 1; Database issue.), downloaded from Biomart (http://www.biomart.org/), were found in 122 of the 128 genes in the atherosclerosis module. We then identified seven transcription factors (TFs) that had LIM domains (reference 11 in the manuscript) or had other known interactions with LDB2 (reference 12 in the manuscript). Using TRANSFAC v 11.2 (reference 13 in the manuscript), we identified a total of 171 known TF binding sites for these TFs, of which 81% were present in the 161 promoters (one or more binding sites), suggesting that in principle these promoters could bind the analyzed transcription factor. To investigate how specific this association may be, we selected 10255 human promoters covering the [-600,-1] region relative to transcription start sites as background. We then compared the combination of any two binding sites (two binding sites are less likely to happen by chance) appearing in the target promoter set and in the background sequences, and calculated fold enrichment for each of the cases. We calculated Bonferroni corrected *P*-values using Fisher's exact right-side test. We found that the combination of binding sites was statistically enriched in the target set relative to the considered background, with enrichment varying from 1.2- to 5-fold (Table S10).

Calculating Statistical Hypergeometric *P*-values in Venn Diagrams

The significance of the overlap in the Venn diagram was calculated by using a statistical hypergeometric *P*-value. The calculation is done assuming no correlations exists in the data, which is not the case, but this is to show how unlikely it is that the selection would happen by chance. The formula is:

$$\sum_{k=7}^{49} \frac{\binom{n_1}{k}\binom{N-n_1}{n_2-k}}{\binom{N}{n_2}},$$

where N =15 042 RefSeq transcripts used in the cluster algorithm from start, n_1 =59 transcripts found in the visceral fat cluster, n_2 =49 transcripts found in the atherosclerotic arterial wall/IMA cluster, and k=number of transcripts in the intersection (here 7 transcripts/genes).

For the intersection of all three gene groups, the formula is extended:

$$\sum_{l=6}^{49} \sum_{k=l}^{49} \frac{\binom{n_1}{k}\binom{N-n_1}{n_2-k}}{\binom{N}{n_2}} \frac{\binom{k}{l}\binom{N-k}{n_3-l}}{\binom{N}{n_3}},$$

where n_3 =55 transcripts found in the carotid plaque cluster and l=number of transcripts in the intersection (here 6 transcripts/genes).
